# Reversal of the Skin Effect in Disordered Non-Hermitian Systems

**DOI:** 10.3390/e28030339

**Published:** 2026-03-18

**Authors:** Xiansheng Zeng

**Affiliations:** School of Physics, Southeast University, Nanjing 211189, China; 220232374@seu.edu.cn; Tel.: +86-131-2754-2268

**Keywords:** quantum walk, non-Hermitian skin effect, modified generalized Brillouin zone, disorder

## Abstract

Non-Hermitian systems under nonreciprocity-induced evolution present an exotic phenomenon, known as the non-Hermitian skin effect. Yet, the control mechanisms and the generalized Brillouin zone in disordered systems have not been fully understood. Here, using Floquet quantum-walk models with disorder, we demonstrate effective control of the direction of the non-Hermitian skin effect in both one- and two-dimensional systems. Once the disorder strength exceeds a critical threshold, the direction of the skin effect is reversed. We develop a modified generalized Brillouin zone theory that correctly predicts skin effect reversal. Furthermore, we also investigate how the direction of the non-Hermitian skin effect depends on the disorder strength in each subsystem of the two-dimensional quantum walk. Our work paves the way for the design of quantum transport devices in quantum simulation platforms.

## 1. Introduction

Disorder and non-Hermiticity profoundly modify the topology and localization of quantum systems, leading to novel quantum states of matter, including disorder-induced topological phases [1,2,3,4,5,6] and unconventional non-Hermitian phenomena [7,8,9,10]. On the one hand, although the introduction of disorder tends to close the energy gap, the disorder can induce topology from a trivial insulator, giving rise to a topological Anderson insulator [6,11,12,13,14]. On the other hand, in a broad class of non-Hermitian systems, bulk wave functions become exponentially localized at the boundaries due to the non-Hermitian skin effect (NHSE). Here, a non-Hermitian system refers to a system whose effective Hamiltonian or evolution operator is non-Hermitian, typically arising from gain, loss, or nonreciprocal couplings. The NHSE denotes the macroscopic boundary accumulation of eigenstates under open boundary conditions, which has no Hermitian counterpart. The NHSE was first identified in nonreciprocal lattice models [15,16,17,18,19,20,21,22], later formulated within the framework of non-Bloch band theory [23,24,25,26,27], and subsequently demonstrated in various experimental platforms [6,8,28,29,30,31].

In discrete-time quantum walk implementations, disorder can naturally arise from fluctuations in the coin rotation angles, imperfect phase modulation, or position-dependent variations introduced by experimental noise. Such randomness is inevitable in realistic optical and atomic platforms [32,33,34,35]. To account for these effects, it is therefore necessary to incorporate disorder into the theoretical model and examine its influence on the NHSE.

The generalized Brillouin zone (GBZ) theory, characterized by non-Bloch waves, provides a powerful framework for characterizing the NHSE in translationally symmetric systems [36,37]. In particular, in clean lattices, the GBZ construction successfully predicts not only the existence of the NHSE but also the direction of boundary accumulation through the spectral winding structure [38,39,40]. However, the traditional GBZ formalism fundamentally relies on spatial translational symmetry and a well-defined complex Bloch momentum. Once disorder is introduced, translational symmetry is broken and the complex momentum loses its meaning as a good quantum number. As a result, it is unclear whether the GBZ-based prediction of skin-effect direction remains valid in disordered non-Hermitian systems.

In particular, while the presence of NHSE in clean systems is well understood [16,17,41], the robustness and directional controllability of skin accumulation beyond the translationally symmetry limit have not yet been systematically clarified. This leaves an open question regarding how the direction of NHSE can be characterized and regulated in disordered settings.

Disorder is generally unavoidable and difficult to manipulate. However, within our quantum-walk model, the disorder strength can be treated as a tunable parameter [6,42,43]. In this work, we explore the interplay between disorder and non-Hermiticity in quantum walk where disorder and non-Hermiticity can be precisely engineered and controlled [44,45,46,47]. We numerically demonstrate the reversal of the disorder-induced NHSE. This phenomenon can be explained by the modified GBZ theory. Firstly, We investigate the effect of disorder on the direction of the NHSE using a one-dimensional quantum walk. Furthermore, we extend our model to two-dimensional quantum walk to explore the disorder-induced NHSE, where tuning the disorder strength between the upper and lower chains enables control over the skin effect. This work not only uncovers the reversal of the disorder-induced skin effect in disordered non-Hermitian systems, but also establishes a feasible route to explore such phenomena.

## 2. Reversal of the NHSE in One-Dimensional Disordered Quantum Walk

### 2.1. Non-Unitary Quantum Walk

**Figure 1:**
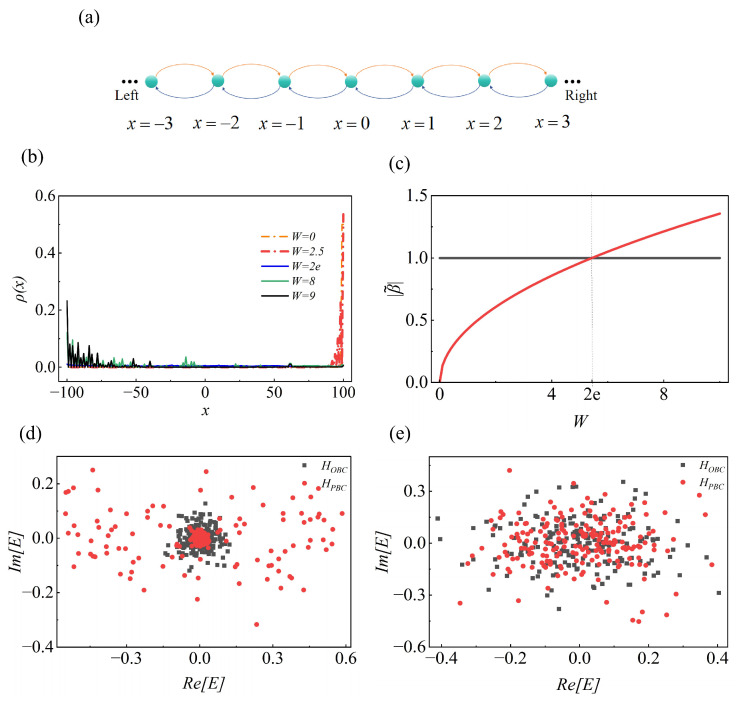
(**a**) The diagram of one-dimensional quantum walk. The walker evolves to the right (left) when the coin state is |0〉 (|1〉), represented by orange (blue) arrow. (**b**) Occupation of the eigenstates of $U_{0}$ under different disorder strengths ($\theta_{1}=0.3\pi ,\theta_{2}=0.8\pi$). (**c**) The modified GBZ $|\tilde{\beta }|$ versus disorder strength W. The conventional Brillouin zone (BZ) corresponding to |β|=1, where the GBZ reduces to the unit circle and the non-Hermitian skin effect disappears. (**d**,**e**) The real part (Re[E]) versus the imaginary part (Im[E]) of the eigenvalue of the operator $U_{0}$ for different disorder strengths with (**d**): W=1, (**e**): W=9.

We first consider a one-dimensional quantum walk, as illustrated in Figure 1a, which is governed by

(1) $$U_{0}=R\left(\theta_{2}\right)MS_{x}R\left(\theta_{1}\right).$$

Here, the shift operator is defined as $S_{x}=\sum_{x}|x-1\rangle \langle x|⊗|0\rangle \langle 0|+|x+1\rangle \langle x|⊗|1\rangle \langle 1|$, where |0〉 (|1〉) serves as the basis of the coin state. The non-Hermiticity is induced by the gain-loss operator $M=\sum_{x}|x\rangle \langle x|⊗$ $\left(\begin{array}{cc} \gamma /2 & 0\\ 0 & 1 \end{array}\right)$, where γ∈[−W,W], *W* represents the disorder strength. The coin operator is $R\left(\theta \right)=\sum_{x}|x\rangle \langle x|⊗$ $\left(\begin{array}{cc} \cos\theta & -\sin\theta \\ \sin\theta & \cos\theta \end{array}\right)$. The dynamic of the quantum walk can be regarded as $|\psi_{t}\rangle =U_{0}^{t}|\psi_{0}\rangle$, the initial state is $|\psi_{0}\rangle =|0\rangle ⊗|0\rangle$. While the NHSE is typically linked to nonreciprocity [23,28,48], disorder can serve as a resource to control it. By analyzing the occupation of eigenstates, we investigate the impact of disorder on the NHSE in quantum walks. The occupation is defined as $\rho \left(x\right)=\frac{\sum_{n}\langle \psi_{n}\left(x\right)|\psi_{n}\left(x\right)\rangle }{2N}$, where *N* denotes the quantum-walk lattice (N=100), $|\psi_{n}\rangle$ denotes the *n*-th eigenstate of $U_{0}$. In Figure 1b, we present the eigenstate occupation of the evolution operator $U_{0}$ under different disorder strengths. We perform 50 random realizations for each *W* and then take the average. The disorder γ follows a uniform distribution given *W*. When *W* is small, the average occupation ρ(x) accumulates near the right boundary, where many eigenstates are exponentially localized, giving rise to right-localized NHSE. When *W* becomes large, the NHSE reverses its direction, and further increasing *W* strengthens the localization of the skin modes.

Although our framework is formulated in terms of a discrete-time (Floquet) quantum walk rather than a time-independent Hamiltonian, the gain/loss operator introduces non-unitary evolution that effectively leads to asymmetric amplification and attenuation during propagation. This mechanism plays a role analogous to non-reciprocal hopping in non-Hermitian tight-binding models and can similarly result in boundary accumulation of eigenstates under open boundary conditions.

### 2.2. Modified GBZ Theory

In disordered non-Hermitian systems, the absence of translational symmetry challenges the conventional bulk-boundary correspondence (BBC), raising a difficulty to the applicability of GBZ theory [15,30,31,41]. Here, we employ the modified GBZ theory [49,50,51]. Within this framework, we obtain the disorder NHSE and further elucidate the mechanism underlying the disorder-induced reversal of the NHSE. In this study, we consider a one-dimensional quantum-walk system. For the quantum walk, we expand the evolution operator $U_{0}$ as

(2) $$U_{0}=\sum_{x}|x\rangle \langle x+1|⊗A_{1}+|x+1\rangle \langle x|⊗A_{2},$$

with

(3) $$A_{1}=R\left(\theta_{1}\right)P_{0}MR\left(\theta_{2}\right),$$

(4) $$A_{2}=R\left(\theta_{1}\right)P_{1}MR\left(\theta_{2}\right).$$

Here, $P_{0}=|0\rangle \langle 0|$ ($P_{1}=|1\rangle \langle 1|$) are projectors. We write the bulk-state ansatz as $|\psi \rangle =\sum_{x}\beta^{x}|x\rangle ⊗|\phi \rangle$. From the eigen equation $U_{0}|\psi \rangle =\lambda |\psi \rangle$ (λ is the eigenvalue), we have

(5) $$A_{1}\beta +A_{2}\frac{1}{\beta }-\lambda =0,$$

(6) $$\beta^{2}+C\left(\lambda ,\theta_{1},\theta_{2}\right)\beta +\frac{\gamma }{2}=0,$$

By sorting the solutions as $\left|\beta_{1}\right|\leq \left|\beta_{2}\right|$ and imposing the condition $\left|\beta_{1}\right|=\left|\beta_{2}\right|$, we obtain $\left|\beta \right|=\sqrt{\frac{\gamma }{2}}$. For the disordered quantum-walk system,

(7) $${\tilde{\beta }}^{2N}=\prod_{x=1}^{N}\frac{\gamma_{x}}{2}.$$

For $\gamma_{x}\in \left[-W,W\right]$, $\gamma_{x}$ representing the disorder at position *x*. The renormalized β can be obtained as follows

(8) $$\ln(|\tilde{\beta }\left|\right)=\lim_{n\to \infty }\frac{1}{2n}\left[\sum_{x=1}^{n}\ln\left(\frac{|\gamma_{x}|}{2}\right)\right]$$

(9) $$=\int_{-W}^{W}\ln\left(\frac{\left|x\right|}{2}\right)\frac{dx}{4W}$$

(10) $$=\frac{1}{2}\left[\ln\left(\frac{W}{2}\right)-1\right].$$

We have

(11) $$\left|\tilde{\beta }\right|=\sqrt{\frac{W}{2e}}.$$

In Figure 1c, we plot $|\tilde{\beta }|$ as a function of *W*. The sign of $\left|\tilde{\beta }\right|-1$ specifies the direction of the skin effect. We find the critical value W=2e (*e* is the base of the natural logarithm). As shown in Figure 1c, $|\tilde{\beta }|$ provides an accurate characterization of the change in the NHSE direction. For W<2e, $\left|\tilde{\beta }\right|-1$ decreases with increasing *W* and remains negative. When W>2e, the sign of $\left|\tilde{\beta }\right|-1$ changes, signaling a reversal of the NHSE direction. For larger *W*, the disorder-enhanced NHSE becomes more pronounced, consistent with Figure 1b.

To distinguish the NHSE from a simple biased drift induced by non-unitary amplification, we further compare the spectra of the evolution operator $U_{0}$ under periodic and open boundary conditions. A characteristic feature of the NHSE is the strong sensitivity of the spectrum to boundary conditions, reflecting the breakdown of the conventional bulk–boundary correspondence in non-Hermitian systems. As shown in Figure 1d,e, the eigenvalue spectrum under OBC is significantly deformed compared with that under PBC, accompanied by boundary accumulation of eigenstates. Within the generalized Brillouin zone description, this behavior is captured by the parameter $|\tilde{\beta }|$, whose magnitude determines the spatial amplification or attenuation of eigenmodes and thus the direction of skin accumulation.

**Figure 2:**
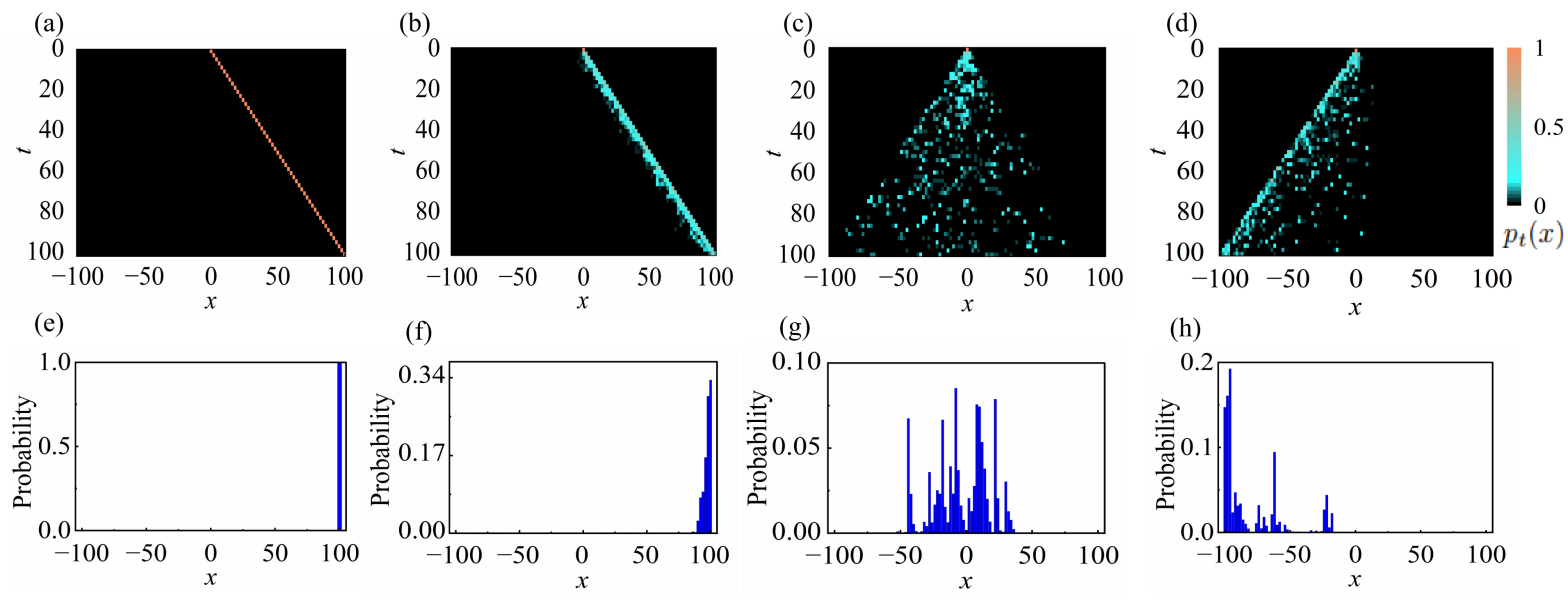
Simulation results of dynamical evolution in non-Hermitian quantum walk with varying disorder strengths. (**a**–**d**) The normalized spatial probability distributions $p_{t}\left(x\right)$. Here we choose x=0,m=0, $|\psi_{0}\rangle =|0\rangle ⊗|0\rangle$. Other parameters are set to $\theta_{1}=0.3\pi$ and $\theta_{2}=0.8\pi$. The quantum-walk simulation results in panels (**a**–**d**) correspond to non-Hermitian quantum walk with disorder strengths of (**a**) W=0 (**b**) W=2.5 (**c**) W=2e (d) W=9. (**e**–**h**) The probability distribution after the last time step t=100, corresponding to non-Hermitian quantum walk with disorder strengths of (**e**) W=0 (**f**) W=2.5 (**g**) W=2e (**h**) W=9.

Furthermore, we numerically simulate the probability distribution after 100 quantum-walk steps. We consider the normalized spatial distribution $p_{t}\left(x\right)=\sum_{m=0,1}\langle \psi_{t}^{x,m}|\psi_{t}^{x,m}\rangle$, where $|\psi_{t}^{x,m}\rangle$ is the final state at position *x* and coin state *m*, and *t* denotes the number of steps in the quantum walk. As shown in Figure 2a–h, the NHSE emerges for W<2e. When *W* exceeds the critical value, the skin effect direction reverses, and further increasing *W* enhance the localization of the skin modes. This is consistent with the predictions obtained from our modified GBZ predictions.

**Figure 3:**
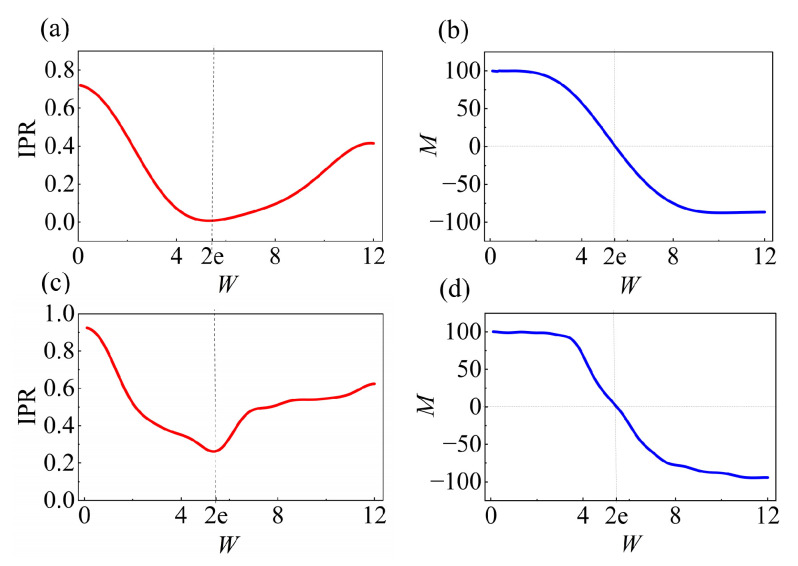
(**a**) The variation in IPR of the eigenstates of the quantum-walk operator $U_{0}$ as a function of *W*. (**b**) The variation in the MP of the eigenstates of the quantum-walk operator $U_{0}$ as a function of *W*. (**c**) IPR of the quantum walk after 100 steps. Here, we choose x=0,m=0, $|\psi_{0}\rangle =|0\rangle ⊗|0\rangle$. Other parameters are set to $\theta_{1}=0.3\pi$ and $\theta_{2}=0.8\pi$. (**d**) MP of the quantum walk after 100 steps. The initial state and parameter values are identical to those used in panel (**c**).

Figure 3 shows the eigenstates of the quantum-walk operator $U_{0}$, along with the evolution of the inverse participation ratio (IPR) [52,53] and the mean position (MP) [54,55] after 100 steps of the quantum walk governed by $U_{0}$. As a function of *W*, the expressions for the IPR and MP are given by

(12) $$\mathrm{IPR}=\frac{1}{4N^{2}}\sum_{n=1}^{2N}\sum_{x=1}^{2N}{\left|\psi_{n}\left(x\right)\right|}^{4},$$

(13) $$MP=\frac{1}{4N^{2}}\sum_{n=1}^{2N}\sum_{x=1}^{2N}x{\left|\psi_{n}\left(x\right)\right|}^{2}.$$

From the variation in the IPR with the disorder strength *W* (Figure 3a,c), we find that for small *W* the quantum walk exhibits a strong NHSE, resulting in large IPR values. As *W* increases toward 2e, the skin effect is suppressed, the quantum walk enters an extended phase with minimal localization, and the IPR reaches its global minimum. For W>2e, the skin effect reverses and evolves into a disorder-enhanced skin effect, with the localization strength increasing continuously with *W*, resulting in a characteristic V-shaped dependence of the IPR with a minimum value at W=2e.

From the variation in the mean position MP with disorder strength *W* (Figure 3b,d), we observe that for small *W* the quantum walk exhibits a strong NHSE near the right boundary, resulting in large values of MP. As *W* increases toward the critical value, the skin effect disappears and the quantum walk exhibits an extended behavior with minimal localization. For *W* beyond the critical point, the skin effect reverses its direction and evolves into a disorder-enhanced regime, leading to the relocalization of eigenstates at the left boundary. Consequently, MP increases monotonically with *W*, with the critical disorder strength marking the crossover between the two regimes.

All the above numerical results are in excellent agreement with the theoretical predictions based on the modified GBZ, confirming that the modified GBZ can accurately predict the emergence of skin effect in disordered non-Hermitian quantum walks, as well as disorder-induced reversal of its direction.

## 3. Reversal of the Non-Hermitian Skin Effect in Two-Dimensional Quantum Walk

**Figure 4:**
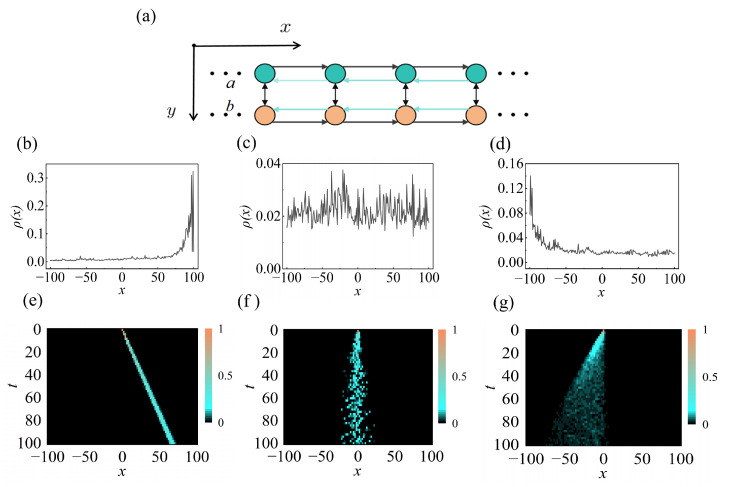
(**a**) The diagram of two-dimensional quantum walk. (**b**) The probability occupation averaged over all eigenstates in a two-dimensional quantum walk with a disorder strength of $W_{1}=W_{2}=1$. (**c**) The same as (**a**), but $W_{1}=W_{2}=2e$. (**d**) The same as (**a**), but $W_{1}=W_{2}=9$. (**e**) The normalized spatial probability distributions obtianed from the dynamical evolution. We choose x=0,m=0, $|\psi_{0}\rangle =\left(|a,0\rangle ⊗|0\rangle +|b,0\rangle ⊗|0\rangle \right)/\sqrt{2}$. (**f**) The same as (**e**), but $W_{1}=W_{2}=2e$. (**g**) The same as (**e**), but $W_{1}=W_{2}=9$. Other parameters are set to $\theta_{1}=0.2\pi ,\theta_{2}=0.3\pi ,\theta_{3}=0.8\pi$.

Previously, we analyzed a one-dimensional quantum walk, in which the probability distribution spreads along a single spatial dimension at each step. In higher dimensions, the probability distribution can propagate along different directions depending on the chosen protocol. Here, we consider a specific type of two-dimensional quantum walks, namely the ladder quantum walk, which is realized by coupling two one-dimensional walks, as illustrated in Figure 4a. We consider the ladder quantum walk

(14) $$U=C\left(\theta_{1}\right)S_{2}C\left(\theta_{2}\right)S_{4}C\left(\theta_{3}\right)MC\left(\theta_{3}\right)S_{3}C\left(\theta_{2}\right)S_{1}C\left(\theta_{1}\right).$$

The shift operators are given by

(15) $$S_{1}=\sum_{x}\sum_{y\in \left\{a,b\right\}}|y,x\rangle \langle y,x|⊗|1\rangle \langle 1|+|y,x+1\rangle \langle y,x|⊗|0\rangle \langle 0|,$$

(16) $$S_{2}=\sum_{x}\sum_{y\in \left\{a,b\right\}}|y,x\rangle \langle y,x|⊗|0\rangle \langle 0|+|y,x-1\rangle \langle y,x|⊗|1\rangle \langle 1|,$$

(17) $$S_{3}=\sum_{x}\sum_{y\neq y^{\prime }\in \left\{a,b\right\}}|y,x\rangle \langle y^{\prime },x|⊗|0\rangle \langle 0|+|y,x\rangle \langle y,x|⊗|1\rangle \langle 1|,$$

(18) $$S_{4}=\sum_{x}\sum_{y\neq y^{\prime }\in \left\{a,b\right\}}|y,x\rangle \langle y^{\prime },x|⊗|1\rangle \langle 1|+|y,x\rangle \langle y,x|⊗|0\rangle \langle 0|.$$

The coin rotation operator is given by $C\left(\theta \right)=\sum_{x}\sum_{y\in \left\{a,b\right\}}|y,x\rangle \langle y,x|⊗e^{-i\theta \sigma_{2}/2}$. Here, *x* labels the position of the walker, and y=a,b denote the two legs of the ladder. The non-Hermiticity is introduced by the gain-loss operator $M=\sum_{x}\sum_{y\in \left\{a,b\right\}}|y,x\rangle \langle y,x|⊗\left(\frac{\gamma_{a/b}}{2}|1\rangle \langle 1|+|0\rangle \langle 0|\right)$ ($\gamma_{a}\in \left[-W_{1},W_{1}\right],\gamma_{b}\in \left[-W_{2},W_{2}\right]$).

Following the approach in the previous section, we obtain the critical value $W_{1}=W_{2}=2e$ (see Equation (25)). At finite disorder strength, the eigenstates of the quantum walk are localized toward the right boundary (Figure 4b). Upon further increasing the disorder strength to the critical value, the skin effect is suppressed (Figure 4c). When the disorder strength exceeds the critical value, the skin effect direction reverses, as shown in Figure 4d. To verify these results, we simulate the evolution of the quantum-walk probability distributions (Figure 4e–g). These results demonstrate the disorder-induced reversal of the skin effect in the quantum-walk dynamic. We consider the normalized spatial probability distribution $P_{t}\left(x\right)=\sum_{y\in \left\{a,b\right\};m=0,1}\langle \psi_{t}^{x,y,m}|\psi_{t}^{x,y,m}\rangle$, where $|\psi_{t}^{x,y,m}\rangle$ denotes the normalized final state.

**Figure 5:**
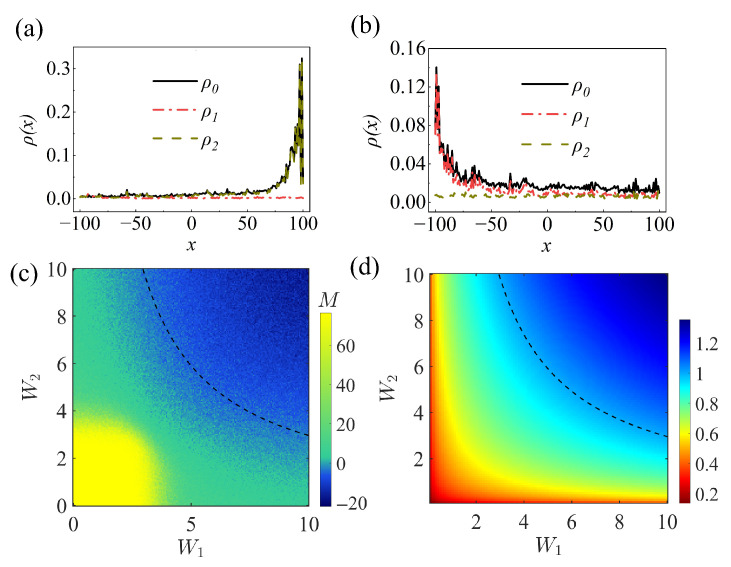
(**a**) Occupations of eigenstates for the quantum walk with disorder strength $W_{1}=W_{2}=1$. Here, $\rho_{0}$ denotes the occupation of all eigenstates, while $\rho_{1}$ and $\rho_{2}$ correspond to the occupations on the first and second chains, respectively. (**b**) The same as (**a**) but $W_{1}=W_{2}=9$. (**c**) Color plot of the mean position (MP) in a two-dimensional quantum walk after a 100-step quantum walk, showing the distribution as a function of the disorder strengths in the upper and lower chains. Yellow (blue) indicates right- (left-) skin of the quantum walk. MP=0 indicates that the quantum walk is in an extended state. (**d**) Theoretical diagram in terms of the color contour of the numerically evaluated modified GBZ along the *x* direction, with $\theta_{1}=0.2\pi$, $\theta_{2}=0.3\pi$ and $\theta_{3}=0.8\pi$. The red (blue) region corresponds to the $\left|{\tilde{\beta }}_{x}\right|-1>0\left(<0\right)$.

To further explore the impact of the disorder strengths on the upper and lower chains in the skin effect reversal of the two-dimensional quantum walk, we analyze the eigenstate occupations of the two chains. At finite disorder strength, the eigenstates of the quantum walk are localized toward the right boundary. By analyzing the eigenstate-averaged occupations on the first and second chains, we indicate that the *b* chain dominates in this regime, leading to an overall right-direction NHSE (Figure 5a). Upon further increasing the disorder strength beyond the critical value, the direction of the NHSE is reversed. A similar analysis of the average eigenstate occupations on the two chains shows that the *a* chain now becomes dominant, leading to an overall left-direction skin effect (Figure 5b). These results reveal the crucial role of disorder strength in determining the direction of the NHSE in a two-dimensional quantum walk and clarify the underlying mechanism.

Notably, the skin effect reversal in the two-dimensional ladder quantum walk arises from the distinct disorder strengths applied to the upper and lower chains. As shown in Figure 5c, we present the relationship between the disorder strength on the two chains and the resulting skin direction, where the yellow (blue) regions correspond to right (left) skin effect.

### Modified GBZ Along the x Direction

To clarify the relation between the skin direction in the two-dimensional quantum walk and the disorder strengths on the two chains, we analyze the modified GBZ along the *x* direction. We expand the evolution operator *U* as

(19) $$U=\sum_{x}\sum_{y\in \{a,b\}}|x\rangle \langle x+1|⊗B_{1}+|x+1\rangle \langle x|⊗B_{2}+|x\rangle \langle x|⊗B_{3},$$

with

(20) $$\begin{array}{cc} B_{1} & =C\left(\theta_{1}\right)\left(P_{1a}+P_{1b}\right)C\left(\theta_{2}\right)\left(P_{0a}+P_{0b}+P_{1a}+P_{1b}\right)\\ & C\left(\theta_{3}\right)M\left(P_{0a}+P_{0b}+P_{1a}+P_{1b}\right)C\left(\theta_{2}\right)\left(P_{1a}+P_{1b}\right)\\ & C\left(\theta_{2}\right)C\left(\theta_{1}\right), \end{array}$$

(21) $$\begin{array}{cc} B_{2} & =C\left(\theta_{1}\right)\left(P_{0a}+P_{0b}\right)C\left(\theta_{2}\right)\left(P_{0a}+P_{0b}+P_{1a}+P_{1b}\right)\\ & C\left(\theta_{3}\right)M\left(P_{0a}+P_{0b}+P_{1a}+P_{1b}\right)C\left(\theta_{2}\right)\left(P_{0a}+P_{0b}\right)\\ & C\left(\theta_{2}\right)C\left(\theta_{1}\right), \end{array}$$

(22) $$\begin{array}{cc} B_{3} & =C\left(\theta_{1}\right)\left(P_{0a}+P_{0b}\right)C\left(\theta_{2}\right)\left(P_{0a}+P_{0b}+P_{1a}+P_{1b}\right)\\ & C\left(\theta_{3}\right)M\left(P_{0a}+P_{0b}+P_{1a}+P_{1b}\right)C\left(\theta_{2}\right)\left(P_{1a}+P_{1b}\right)\\ & C\left(\theta_{2}\right)C\left(\theta_{1}\right). \end{array}$$

Here, $P_{0a}\left(P_{0b}\right)$ and $P_{1a}\left(P_{1b}\right)$ denote the projectors $P_{0}=|0\rangle \langle 0|$ and $P_{1}=|1\rangle \langle 1|$ on the *a* (*b*) chain, respectively. We write the state along *x* direction as $|\psi_{x}\rangle =\sum_{x}\beta^{x}|x\rangle ⊗|\phi \rangle$. As in the previous one-dimensional quantum walk, we have

(23) $$\beta_{x}^{2}+C\left(\lambda ,\theta_{1},\theta_{2},\theta_{3}\right)\beta_{x}+\sqrt{\frac{\gamma_{a}\gamma_{b}}{4}}=0,$$

(24) $$\beta_{x}={\left(\frac{\gamma_{a}\gamma_{b}}{4}\right)}^{\frac{1}{4}},$$

(25) $$\left|{\tilde{\beta }}_{x}\right|=\sqrt{\frac{{\left(W_{1}W_{2}\right)}^{1/2}}{2e}}.$$

Next, we plot a heatmap of the modified GBZ along *x* versus the disorder strengths of the upper and lower chains. As shown in Figure 5d, the sign of $\left|{\tilde{\beta }}_{x}\right|-1$ determines the direction of the skin effect in the quantum walk. A negative value ($\left|{\tilde{\beta }}_{x}\right|-1<0$) corresponds to a right-skin accumulation, whereas a positive value ($|{\tilde{\beta }}_{x}|-1>0$) corresponds to a left-skin accumulation. In Figure 5c,d, we plot the boundary defined by MP=0 (dashed black curve in Figure 5c) together with that defined by $\left|{\tilde{\beta }}_{x}\right|-1=0$ (dashed black curve in Figure 5d). The modified GBZ we calculated along the *x* direction can accurately predict the skin effect direction in the ladder quantum walk. Furthermore, tuning the disorder strength between the two chains offers a practical means to control this direction.

## 4. Conclusions

In realistic experimental implementations, the dynamics of quantum systems may also be influenced by external environments. Such environments can introduce dissipation and decoherence processes that are commonly described within Markovian or non-Markovian open-system frameworks. Previous studies have shown that environmental effects can significantly modify the transport and localization properties in quantum walks and non-Hermitian systems [56,57,58]. In many cases, effective non-Hermitian descriptions can emerge from the coupling between the system and its environment, where gain and loss processes lead to non-unitary evolution.

In this work, we design a one-dimensional lattice quantum-walk scheme to simulate disordered non-Hermitian systems. Our approach exploits quantum walks to mimic the quantum dynamics of non-Hermitian systems. Within this framework, we demonstrate disorder-induced reversal of the NHSE. Furthermore, in discrete-time nonunitary quantum-walk dynamics, we confirm that the modified GBZ can accurately predict the emergence of NHSE in disordered non-Hermitian systems. As a complementary extension, we introduce a ladder quantum-walk to illustrate the reversal of the skin effects. This study reveals the intrinsic connection among the orientation of the NHSE, disorder strength, and subsystem characteristics, providing new perspectives and potential pathways for advancing disordered non-Hermitian physics and its applications.

## Data Availability

The data presented in this study are available on request from the corresponding author. The data are not publicly available due to privacy restrictions.

## Abbreviations

The following abbreviations are used in this manuscript:

NHSE	Non-Hermitian Skin Effect
GBZ	Generalized Brillouin Zone
BBC	Bulk-Boundary Correspondence
IPR	Inverse Participation Ratio
